# Hepatitis C Virus Infection in Hemodialysis Patients in the Era of Direct-Acting Antiviral Treatment: Observational Study and Narrative Review

**DOI:** 10.3390/medicina60122093

**Published:** 2024-12-21

**Authors:** Ioana Adela Ratiu, Adelina Mihaescu, Nicu Olariu, Cristian Adrian Ratiu, Bako Gabriel Cristian, Anamaria Ratiu, Mirela Indries, Simona Fratila, Danut Dejeu, Alin Teusdea, Mariana Ganea, Corina Moisa, Luciana Marc

**Affiliations:** 1 Faculty of Medicine and Pharmacy, University of Oradea, 1st December Square 10, 410073 Oradea, Romania; ioana.ratiu@didactic.uoradea.ro (I.A.R.); bako_gabriel@uoradea.ro (B.G.C.); mirela.indries@didactic.uoradea.ro (M.I.); sfratila@uoradea.ro (S.F.); ddejeu@uoradea.ro (D.D.); mganea@uoradea.ro (M.G.); 2 Nephrology Department, Emergency Clinical Hospital Bihor County, 12 Corneliu Coposu Street, 410469 Oradea, Romania; 3 Department of Internal Medicine II—Division of Nephrology, “Victor Babeș” University of Medicine and Pharmacy, 300041 Timisoara, Romania; mihaescu.adelina@umft.ro (A.M.); olariu.nicu@umft.ro (N.O.); marc.luciana@umft.ro (L.M.); 4 Center for Molecular Research in Nephrology and Vascular Disease, Faculty of Medicine, “Victor Babeș” University of Medicine and Pharmacy, 300041 Timișoara, Romania; 5 Dentistry Department, Faculty of Medicine and Pharmacy, University of Oradea, 1st December Square 10, 410073 Oradea, Romania; ratiu_cristian@uoradea.ro; 6 Faculty of Dentistry, University of Medicine and Pharmacy “Iuliu Hatieganu” Cluj-Napoca, Victor Babeș 8, 400347 Cluj-Napoca, Romania; ratiu.anamaria@elearning.umfcluj.ro; 7 Infectious Diseases Department, Emergency Clinical Hospital, 410167 Bihor County, Romania; 8 Faculty of Environmental Protection, University of Oradea, G-ral Magheru 27, 410087 Oradea, Romania; ateusdea@uoradea.ro

**Keywords:** hepatitis C virus, hemodialysis, direct-acting antivirals (DAAs), comorbidities, malignancies

## Abstract

*Background and Objectives:* Hepatitis C virus (HCV) infection is a major global public health concern, particularly in hemodialysis (HD) patients. This study aims to evaluate the demographic, clinical, and laboratory characteristics of HCV-positive patients undergoing HD and assess the long-term impact of direct-acting antivirals (DAAs) on patient outcomes. Moreover, a narrative review aims to summarize the current knowledge regarding HCV treatment in HD patients. The search in the PubMed, Google Scholar, and Scopus databases identified 48 studies relevant to our topic, 18 regarding clinical history and 29 related to HCV treatment. *Methods*: A retrospective analysis was performed on 165 HD patients from Bihor County HD centers, Romania, between 2014 and 2024. The cohort was divided into two groups: 54 patients who tested positive for HCV and 111 controls who were HCV-negative. Data collected from GPs included demographic information, comorbidities, laboratory parameters, and psychological assessments. Outcomes were evaluated at over 5 years after DAA treatment. A literature review was conducted using PubMed and Google Scholar to identify relevant studies on HCV in HD patients from 1989 to 2024. *Results:* Laboratory results showed similar parameters across groups, except for lower serum cholesterol levels in the HCV-positive DAA-treated group vs. HCV-positive non-treated ones (155.607 mg% vs. 170.174 mg%, *p* = 0.040) and increased ALT levels when comparing the same groups (29.107 vs. 22.261, *p* = 0.027), whereas comorbidities did not differ significantly. The incidence of malignancies was significantly higher among HCV-positive compared to HCV-negative patients (20.3% vs. 8.1%, *p* = 0.023), mainly among those treated with DAAs, highlighted by the multivariate analysis. Cardiovascular disease remains the leading cause of mortality regardless of HCV status or the use of antiviral therapy. Psychological assessments revealed more severe depression in HCV-positive patients compared to their HCV-negative counterparts. *Conclusions:* HCV infection in the hemodialysis population typically follows a subclinical course. At over five years after DAA therapy, the results indicate a stabilization of the liver function and the absence of major complications. However, the incidence of malignancies remains high in HCV-positive patients.

## 1. Introduction

HCV infection is a significant public health challenge on a global scale, with an overall prevalence ranging between 2.5 and 2.8% [1,2]. The average prevalence of HCV infection in Europe is 1.8%, and 72% of the seropositive population is infected. Six genotypes are identified, numbered from 1a/b to 6, the genotype 1b being recognized as the wild-type virus, linked to increased incidence and treatment resistance. Genotype 1 is the most prevalent (70% in Central Europe, 55.1% in Western Europe, and 68.1% in Eastern Europe) [3,4]. Romania has been recognized as the country with the greatest prevalence of HCV in Europe but with a decreasing trend over the last decades (4.9% in 1990, 3.23% in 2010, and a more recent estimate of 2.7%) [5,6].

HCV, a member of the Flaviviridae family, is an RNA virus characterized by hepatic tropism and hematogenous transmission. An effective HCV vaccination is yet to be developed due to the structural heterogeneity imparted by the N-terminal hypervariable region.

HCV may be detected by several methods: (1) indirectly, by the presence of HCV antibodies through enzymatic reaction (EIA or ELISA) or light emission (chemiluminescent assay), rapid immunoassay tests, and recombinant immunoblot analysis (RIBA); (2) nucleic acid testing (NAT) via polymerase chain reaction (PCR) techniques, including reverse transcriptase PCR (RT-PCR); and (3) detection of the HCV core antigen, either alone or in conjunction with other HCV proteins [7]. Each diagnostic method has its limitations: antibodies only appear in the serum 2–6 months after the viral infection; the presence of antibodies does not correlate with disease activity, since they may remain positive after viral clearance, or they may be negative in immunocompromised patients [8]. The latest generation of immunoassays, specifically the fourth generation of tests, simultaneously detects HCV capsid antigen and antibodies to the core, NS3, NS4, and NS5 virus sections. These tests have decreased the HCV detection window period by 17 days compared to conventional assays [7]. Genomic amplification techniques are considered the “gold standard” for detecting active HCV replication, with a detection limit of 10–20 IU/mL. They become positive one week after exposure and at least 4–6 weeks before seroconversion, the drawback being the high cost [9]. HCV core antigen detection techniques are less expensive and become positive approximately 40–50 days earlier than the current third-generation anti-HCV screening assays, but their sensitivity is significantly lower than NAT techniques, ranging between 500 and 3000 IU/mL [10].

In patients with end-stage renal disease (ESRD), the most common renal replacement therapy is extracorporeal dialysis [11]. Given the hematogenous transmission of HCV, it is critically important to detect HCV in patients undergoing HD. The 2022 KDIGO (Kidney Disease Improving Global Outcomes) guidelines recommend the use of NAT as the diagnostic method in HD patients, or alternatively, immunoassay followed by NAT if positive [12]. However, numerous studies suggest that HCV core antigen detection for the screening and follow-up of patients on HD could increase diagnostic accuracy with reasonable costs [8].

The prevalence of HCV in patients undergoing HD varies over time, across countries, and throughout regions. In 2004, the DOPPS (Dialysis Outcomes and Practice Patterns Study) reported a 13.5% prevalence, with significant variations from 2.6% in the UK to 22.9% in Spain, while Japan and the US exhibited similar rates of approximately 14%. In this context, HCV testing was conducted quarterly in the UK, biannually in Spain, and annually in Japan, whereas in the US, it was not frequently performed. Consequently, for dialysis units registered from 1996 to 2011, the worldwide prevalence of infection diminished to 9.5% [13]. Meta-analyses estimate the global prevalence of HVC in the HD population ranges from 20.7% to 24.3% [3,4], which is 10-fold higher than in the general population, surpassing even that reported in prisoners [14,15]. The use of direct-acting antivirals (DAAs) with a collaborative care model implemented by gastroenterology and nephrology specialists demonstrates that the battle against HCV within HD units can be successful with multidisciplinary collaboration and adequate funding and is expected to accelerate HCV eradication in the dialysis population. [16]

An evaluation of the distribution of viral genotypes in HD patients reveals that genotype 1b is the most prevalent (33.5%), followed by 1a (22.8%), 3 (8.2%), 2 (6%), 4 (5%), and 6 (2.4%) [3].

The particular risk factors for HCV infection in hemodialysis patients encompass a prolonged duration on HD, younger age, the co-existence of diabetes mellitus, the frequency of blood transfusions received, and the reuse of dialyzers [3].

**Figure 1:**
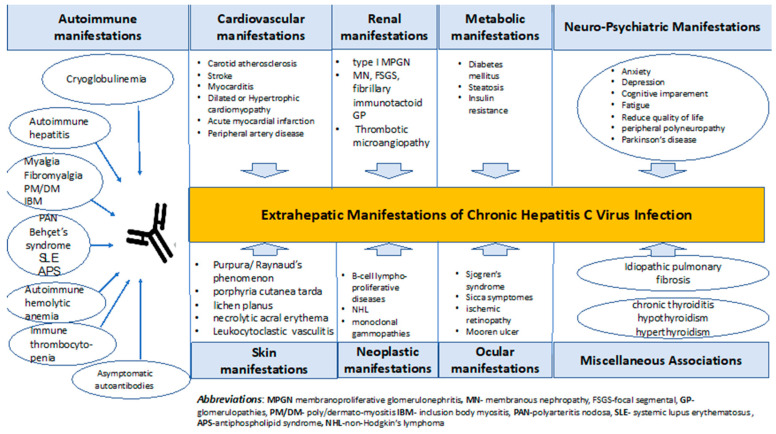
Extrahepatic manifestations of HCV infection [17,18,19].

The clinical manifestation of HCV-infected individuals is markedly heterogeneous. Acute hepatitis may exhibit clinical signs, although chronic hepatitis is frequently silent. The prevalence of cirrhosis is around 10–20% after two decades, while the annual incidence of hepatocellular carcinoma ranges between 1 and 4%. Spontaneous clearance occurs in 25–50% of individuals with symptomatic infections and in 10–15% of individuals with asymptomatic infections [7]. HCV infection is frequently associated with extrahepatic manifestations such as diabetes mellitus, lymphomas, autoimmune thyroiditis, fulminant fibrosing cholangitis, and essential mixed cryoglobulinemia (Figure 1).

An assessment of the natural history of HCV infection in patients on HD is rather challenging due to the multiple comorbidities present in these patients. Some studies indicate the evolution of acute HCV infection in HD patients is similar to the general population [20]. Other studies show that, during HD, viral clearance occurs in asymptomatic patients, while chronic hepatitis presents mild activity, possibly due to the immune alterations induced by the persistent virus [21]. The progression to hepatocellular carcinoma has variable prevalence in the literature, reported to be under 2% [22]. Undoubtedly, the presence of HCV reduces survival in HD patients, related not only to the progression of chronic liver disease to cirrhosis and hepatocellular carcinoma but also to an increased risk of cardiovascular mortality, currently unexplained [20,23,24,25]. Therefore, it is anticipated that direct-acting antiviral treatment for HCV will improve the long-term outcomes of HCV-positive patients in HD.

Using interferon over the years as an antiviral therapy showed a poor tolerability due to adverse effects, particularly when administered to patients that were on kidney transplant lists [26,27,28,29]. Direct-acting antiviral therapy was introduced in 2017. The accessibility of this therapy and the types of antiviral combinations have varied significantly over time, depending on the country. Initially, the DAA regimens examined in individuals with a low glomerular filtration rate (GFR) comprised agents that are cleared by the liver, notably the combinations of NS5A and NS3/4A inhibitors Elbasvir (EBR)/Grazoprevir (GZR) and Glecaprevir (GLE)/Pibrentasvir (PIB). A supplementary regimen metabolized by the liver is the combination of Paritaprevir, Ritonavir, and Ombitasvir with or without Dasabuvir (PrO ± D), achieving sustained virologic response (SVR) rates of 95% in patients with chronic kidney disease CKD G4 to G5 and G5D [30].

The response to DAA treatment in HD patients is influenced as well by the viral genotypes. The studies EXPEDITION 4 and 5 reveal the efficiency of the GLE/PIB combination for all viral genotypes in these patients [31]. The study C-SURFER shows the efficacy of EBR/GZR against genotype 1 in HD, and, although patients with genotype 4 were not included, the beneficial effect of this combination against genotype 4 was extrapolated, similar to the general population [32]. This fact is also confirmed by the study TRIO [33]. The combination sofosbuvir (SOF) with ledipasvir or velpatasvir proved safe and efficient against all viral genotypes.

Sofosbuvir, an NS5B inhibitor with pangenotypic effects, has historically been contraindicated for this patient population due to its primary renal clearance mechanism. SOF’s predominant metabolite, GS-331007, is renally eliminated and accumulates 5- to 20-fold in patients with advanced CKD or undergoing hemodialysis (HD), respectively. However, studies have indicated that the off-label administration of sofosbuvir yielded favorable outcomes, with SVR rates exceeding 95% and no reported adverse effects. In 2019, the US Food and Drug Administration and the European Medicines Agency extended the approval of sofosbuvir as a pangenotypic therapy in HD patients without dose reduction requirements but exclusively in combination therapy [34,35,36,37]. Even so, given the availability of protease inhibitor-containing direct-acting antiviral regimens, there was a reluctance by some practitioners to use SOF-containing regimens in moderate to severe kidney disease.

The combination of sofosbuvir with ledipasvir or velpatasvir proved safe and efficient against all viral genotypes. A suboptimal or inadequate dose of DAA will induce resistance-associated substitutions (RASs). Thus, ledipasvir/sofosbuvir and EBR/GZR induce RASs in genotype 1a and 1b, while sofosbuvir/velpatasvir and GLE/PIB do so in genotype 3.

After DAA treatment, the long-term outcomes of HCV-positive patients undergoing HD remain insufficiently explored. Given the number of HCV-positive patients in our follow-up, we highlighted the primary progressive clinical and laboratory characteristics of these patients. We consider these data valuable for understanding this pathology and its long-term prognosis. These findings may also serve as a basis for future studies and, when corroborated with results from similar studies, can contribute to meta-analyses with a meaningful statistical power regarding the optimal therapeutic management for HD patients.

We divided our work into two parts: first, we presented our personal contribution regarding the evolution of HCV infection in HD patients, and then we reviewed the literature.

Part I—Clinical research—Observational study

## 2. Materials and Methods

This study aimed to assess the long-term outcomes of HD patients infected with hepatitis C virus, including those receiving direct-acting antiviral (DAA) treatment, in a country with a high prevalence of this infection.

We conducted an observational, retrospective, non-interventional study involving 165 patients treated between 2014 and 2024 in HD centers in Bihor County, a region in Western Romania. The study was conducted according to the guidelines of the Declaration of Helsinki and approved by the Ethics Committee of County Emergency Hospital Bihor, Romania (approval number 31131/2024).

Diagnostic criteria:

The chemiluminescent microparticle immunoassays (CMIAs) Abbot system, ARCHITECT analyzer and CLIA Immunoassay (SIEMENS Advia Centaur XP/XPT system) were used until 2020, and afterwards electrochemiluminescence immunoassays (ECLIA) (Roche Diagnostic, Elecsys cobas e immunoassay analyzers) were used to detect the presence of HCV antibodies in 54 of these individuals, who were classified as HCV(+). A total of 111 patients who tested negative for HCV formed the HCV(−)group. According to the standards of dialysis centers in our country, the presence of HCV was assessed at the start of HD treatment and subsequently every six months or as required when a recent HCV infection was suspected. In cases where tests showed unclear results or antiviral therapy was initiated, HCV viremia was additionally evaluated. The antiviral treatment group, HCV(+)DAA-Tr, was represented by 23 patients from those classified as HCV(+), who received the combination of PrO ± D for 12 weeks under the national reimbursement between 2017 and 2019. Inclusion criteria for DAA treatment were any value of RNA-HCV over the detection limit of 15 UI/mL and an F2-F4 fibrosis liver evaluated at biopsy punction or Fibromax. In these patients, HCV viremia was assessed using a PCR-RNA test both prior to and after treatment and the Fibromax test was used to verify the eligibility criteria for inclusion in the national treatment programs. We also included 3 patients who received IFN treatment before starting HD, classified as HCV(+)Ifn-Tr.

Hemodialysis treatment characteristics

Braun Dialog+, Fresenius 4008s, Gambro AK95, AK96, AK 200, and more recently Nipro Surdial X were the hemodialysis machines used during the time. The water purifying stations supplied pure water under 0.1 UFC/mL. The dialyzers were not reused in any of the cases; in fact, dialyzer reuse has no longer been a practice in Romania for more than 30 years. Polysulfone membrane dialyzers (Elisio/Nipro/Japan, FX-Fresenius, Diacap Pro Braun) were used, 1.9–2.1 m^2^, (ultrafiltration coefficient) Kuf 75–82 mL/h/mmHg, gamma-ray sterilization and helix one, 1.8–2.2 m^2^, UF coefficient 53–68 mL/h/mmHg, inline steam sterilization, at 121 degrees Celsius, 15 min. Hemodialysis prescriptions aim to achieve a kT/V value between 1.2 and 1.4 with three HD sessions per week.

Collected data for our patients

Step 1: We included in our study all HCV(+) HD patients, with or without antiviral treatment, and compared them with HCV(−) HD patients. We classified the two groups demographically, including criteria associated with renal replacement therapy: age, sex, duration of hemodialysis, type of vascular access, history of prior transplantation, body mass index (BMI) in association with body surface area (BSA), in order to exclude any form of bias associated with the use of BMI alone. We subsequently examined the primary laboratory parameters associated with kidney and liver conditions: hematological parameters (serum hemoglobin, platelets), cytolytic enzymes (aspartate aminotransferase (AST), alanine aminotransferase (ALT)), alkaline phosphatase, nutritional status indicators (serum albumin, total cholesterol), parameters related to calcium–phosphate balance (intact parathormone (iPTH), calcium, phosphorus, serum bicarbonate), the presence of inflammatory syndrome (C reactive protein—CRP), and the effectiveness of hemodialysis (serum creatinine, eKT/V). We considered the latest laboratory parameters of the patients during the study period, along with the most recent analyses available for those who had passed away. All analyses were conducted in the same laboratory setting for every sample. Finally, we compared the groups for the presence of comorbidities: diabetes mellitus, hypertension, coronary artery disease requiring interventional surgery, incidence of stroke, incidence of neoplasia, age at death, and primary causes of death.

Step2: We compared the HCV(+) group treated with direct-acting antivirals (HCV+ DAA-Tr) with the HCV(−) group, evaluating the same demographic and laboratory parameters as in step 1.

Step 3: We compared the HCV(+) group treated with direct-acting antivirals (HCV(+) DAA-Tr) with untreated HCV(+) patients (HCV(+) nTr).

Step 4: We included all patient categories in the statistical analysis: untreated HCV(+), HCV(+) treated with DAAs (HCV(+) DAA-Tr), HCV(+) treated with interferon (HCV INF-Tr), and HCV(−) patients. The Kruskal–Wallis nonparametric test, Fisher’s exact test, and Wilcoxon test were applied to compare these multiple independent groups simultaneously.

Step 5: Finally, considering the influence of HCV on physical and mental health through multiorgan impairment, we evaluated the impact of HCV on the quality of life of patients on HD. For this purpose, we compiled the most recent results from the questionnaires routinely administered during periodic psychological assessments to our compliant patients, the Beck Depression Inventory (BDI), Perceived Wellness Survey (PWS), Temporal Satisfaction with Life Scale (TSWLS), and Short-Form 36 Health Survey (SF-36).

The Beck Depression Inventory (BDI) (A.T. Beck, [38]), one of the first scales to quantify depression levels, comprises 21 questions, each item evaluated from 0 to 3. The resulting scores for each patient are as follows: 0–9—normal state, 10–15 points—mild depression, 16–23—moderate depression, and 24–63 points—severe depression. The Perceived Wellness Survey (PWS) (T. Adams, J. Bezner, and M. Steinhardt, [39]) assesses well-being as a balance of multiple activities in the subjects’ lives. The scale consists of 36 questions, with 6 subscales that reflect psychological, emotional, social, physical, spiritual, and intellectual dimensions. The result indicate the following: 1–40 below-average well-being, 40–80 moderate well-being, and 80–120 superior well-being. The Temporal Satisfaction with Life Scale (TSWLS) quantifies the level of life satisfaction by comparing the patient’s evaluations across three temporal dimensions, resulting in a global assessment of this parameter. The Short-Form 36 Health Survey (Turner-Boroker, Bartley, Ware [40]) evaluates quality of life across eight dimensions: physical function, role limitations due to physical health, bodily pain, general health perceptions, vitality, social function, role limitations due to emotional problems, and mental health. Interpretation can be conducted for each dimension individually or based on two general scores reflecting physical and mental components. The questions are scored as follows: 1—poor, 2—fair, 3—good, 4—very good, 5—excellent. Scores range from 0 to 100, with higher scores indicating better functioning in their respective areas.

Statistical analysis

Statistical analysis was conducted using Stata/SE 17.0 for Windows (64-bitx86-64) Revision 21.05.2024, copyright 1985–2021 4905 Lakeway Drive, College Station, Texas 77845-4512, USA (state license serial number 401809208832). Continuous variables were assessed using Mann–Whitney and Kruskal–Wallis test, while categorical variables were analyzed with the Chi-square test (χ^2^), Fisher’s exact test, and Wilcoxon pairs test. Continuous variables were reported as means or medians with their respective standard deviations (SDs). To compare multiple independent samples, the Kruskal–Wallis test has been performed, as it uses Dunn’s procedure for multiple pairwise comparisons.

## 3. Results

Analyzing the demographic data of HCV(+) patients compared to HCV(−) patients, no statistically significant differences were observed between the groups in terms of age (66.972 vs. 64.784 years, *p* = 0.403). The male sex predominated in both groups (59.2% vs. 54.9%, *p* = 0.600) (Table 1).

The duration of hemodialysis was similar between the two groups, with an identical median of 7 years (8.85 years for HCV-positive patients and 8.09 years for HCV-negative patients) (Table 1). Although there are no statistical differences between the two groups regarding the percentage of patients with a prior kidney transplant (9.25% vs. 3.6%, *p* = 0.227), when focusing solely on the HCV(Tr) patients, this percentage becomes statistically significant (17.39% vs. 4.5%, *p* = 0.02). The arteriovenous fistula (AVF) remains the primary access route for hemodialysis in both patient categories, with no statistically significant differences (68.5% vs. 75.6%, *p* = 0.431). Also, when comparing nutritional status and ideal weight, we did not record any statistically significant differences between the two groups—ideal weight (69.47 kg vs. 71.55 kg, *p* = 0.27), BMI (25,070 vs. 25,419, *p* = 0.431), or BSA (1.81 vs. 1.82, *p* = 0.3836).

The analysis of biological parameters in the HCV(+) and HCV(−) groups did not reveal any statistically significant differences. Both the mean and median serum hemoglobin levels fell within the target range for patients on hemodialysis, with values of 10,572 g/dL for HCV-positive HD patients compared to 10,662 g/dL for HCV-negative HD patients (*p* = 0.823). The median serum creatinine levels were identical in both groups, at 7.2 mg/dL, and the eKT/V values were similar (1.564 vs. 1.550, *p* = 0.314). There were no statistically differences in serum bicarbonate levels (24,480 vs. 24,273 mEq/L *p* = 0.508), phosphorus levels (4.998 vs. 4.946 mg/dL, *p* = 0.876), total serum calcium (8.913 vs. 8.865 mg/dL, *p* = 0.634), alkaline phosphatase (106.22 vs. 120.83 mg/dL, *p* = 0.23), or iPTH levels (413.096 vs. 4.946 pg/mL, *p* = 0.801). Inflammatory syndrome, assessed through CRP levels, was present in both groups at similar percentages, without statistically significant differences (the median CRP was 15 mg/L for HCV-positive patients vs. 13.3 mg/L for HCV-negative patients, *p* = 0.784). Both the mean and median levels of serum transaminases were within normal limits, with no statistically significant differences between the groups (ALT 25.574 IU/L vs. 22.586 IU/L, *p* = 0.530). The data can be seen in Table 2.

Concerning comorbidities, we did not notice any statistically significant differences in the incidence of hypertension (83.3% vs. 93.6%, *p* = 0.067), diabetes mellitus (20.3% vs. 23.4%, *p* = 0.659), coronary artery disease requiring stent placement (35.1% vs. 36.03%, *p* = 0.914), or stroke (16.6% vs. 10.8%, *p* = 0.289). However, it is noteworthy that patients with HCV infection had a significantly higher incidence of malignancies, with rates of 20.3% compared to just 8.1% in the non-HCV group (*p* = 0.02). No statistically significant differences were seen in age at death, with a nearly similar median age (70 vs. 70.5 years, *p* = 0.079). Table 3 presents the prevalence of major comorbidities identified within the studied HD population.

Patients treated with DAAs were evaluated separately, given the documented presence of fibrosis stage > F2 prior to treatment, which may have a potential long-term negative impact. Nonetheless, laboratory findings in patients who received DAA therapy were comparable to those of HCV-negative patients, with no statistically significant differences identified (Table 4).

When examining the cohort of patients who received anti-HCV DAA treatment (Table 5) the results reveal a poor statistical difference in the association with hypertension, with a prevalence of 78.2% among treated patients compared to 93.6% in untreated patients (*p* = 0.052).

Furthermore, there were no statistically significant differences in the incidence of diabetes mellitus or stroke. On the contrary, patients undergoing anti-HCV treatment showed a higher incidence of malignancies (30.43% vs. 8.1%, *p* = 0.0079), with a significantly lower age at death compared to non-HCV patients (57.63 years vs. 70.76 years, *p* = 0.00098). The leading causes of death in these patients were attributed to malignancies (50% vs. 3.8%, *p* = 0.001) and cardiovascular diseases (37.5 vs. 75%, *p* = 0.02). Table 5 illustrates the comorbidities observed in the population following DAA treatment, emphasizing the changes in their prevalence post-therapy.

When comparing HCV(+) patients treated with DAAs to untreated HCV(+) patients (Table 6), the results indicate that the incidence of malignancies is similar and not influenced by antiviral therapy. However, the age at the time of death was significantly lower among patients who received DAA treatment compared to untreated patients (57.63 vs. 69.893 years, *p* = 0.041).

**Figure 2:**
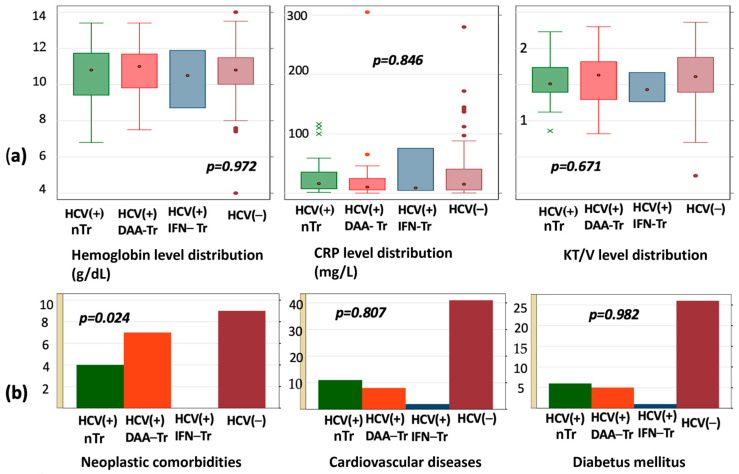
Comparative findings related to HCV status at hemodialysis patients: (**a**) laboratory; (**b**) main commorbidities.

We then performed a multivariate analysis of demographic and laboratory parameters using the Kruskal–Wallis test across the four patient categories: HCV(−), HCV(+) DAA-Tr, HCV(+) IFN-Tr, and HCV(−). This analysis did not reveal any statistically significant differences in the distribution of the evaluated variables (Figure 2, Table 7).

**Figure 3:**
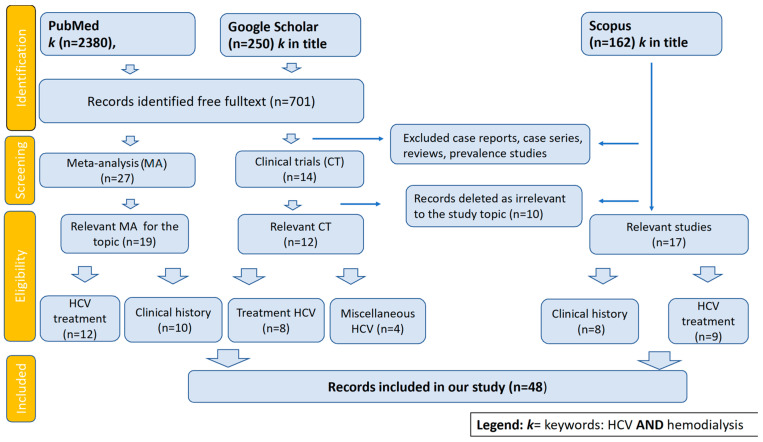
Prisma flowchart for review elaboration.

The evaluation of comorbidities across the four patient categories using the Kruskal–Wallis method revealed no statistically significant differences in their distribution (Table 8, Figure 3).

The assessments for depression, well-being, and quality of life were performed throughout the dialysis course by the psychologist of the department. Data were available for 46 HCV-seropositive patients and 78 HCV-seronegative patients. A comparative evaluation revealed statistically significant differences across all administered tests. The Beck Depression Inventory (BDI) indicated severe depression in both patient groups, with more pronounced symptoms in the HCV-seropositive patients (score 46 vs. 34, *p* = 0.00001). The well-being of the patients, as measured by the Perceived Wellness Survey (PWS), was moderate in both categories, but significantly better in HCV-seronegative patients. Life satisfaction was notably higher among HCV-negative patients compared to their seropositive counterparts. The overall health status, assessed through the Short-Form 36 Health Survey (SF-36), highlighted a moderate level of health among the patients. Table 9 summarizes the tests used to evaluate the psychological conditions associated with HCV infection, providing insights into their prevalence and impact.

Part II—Review from literature

Introduction

Although the prevalence of HCV in the HD population is significant, the long-term progression of HCV seropositive patients, particularly in the DAA era, remains insufficiently evaluated. The use of antiviral treatments, initially interferon-based with or without Ribavirin, and subsequently DAAs, has facilitated the closer monitoring of these patients regarding their immediate response to therapy, but the impact of the monitoring was not extended long-term. This observation prompted a thorough examination of the existing studies specifically addressing patients in chronic HD programs with chronic HCV infections.

This narrative review aimed to present the most relevant literature findings on the long-term progression of patients in maintenance HD, especially concerning the post-antiviral treatment period.

Materials and Methods

(a) *Data Source*

To identify relevant research for this review, we examined the PubMed, Google Scholar, and Scopus databases, spanning the years 1989 to 2024, with the keywords “HCV AND hemodialysis”. Three different investigators collected the data independently, and finally, the results were confronted. The PRISMA statement was consulted throughout this review.

(b) *Inclusion Criteria*

The reports were included if they met the following criteria: (1) They only include the HCV condition in hemodialysis patients. (2) They include at least 4 patients. (3) The studies refer to the progression of HCV infection in HD patients with/without antiviral treatment, rather than estimating the prevalence of HCV infection in these patients. (4) The studies were published in English. We aimed to select meta-analyses or cross-sectional, prospective, retrospective, case–control or cohort studies.

Research Result

Of the 2380 publications identified in PubMed, 693 were accessible as a free full text, allowing their inclusion in our assessment. Using the title-restricted search function in Google Scholar, we identified 250 publications, the majority of which were also located in PubMed. Using the title-restricted key, 162 were selected from Scopus. After excluding the irrelevant studies, 17 were taking into account. We identified 27 meta-analyses directly addressing HCV in hemodialysis, 12 concentrating on anti-HCV treatment, and an additional 10 essentially evaluating the clinical history of HCV within the global hemodialysis population. The research effort provided a singular randomized clinical study concerning the significance of specialized hemodialysis machines for HCV-positive patients, alongside 14 clinical studies centered on anti-HCV therapy in individuals receiving hemodialysis.

**A. META-ANALYSES REGARDING ANTI-HCV TREATMENT**

(a) Interferon-based treatment. The main meta-analyses regarding anti-HCV treatment, listed in chronological order of publication, are presented in Table 10.

A metanalysis of over 20 studies (Gordon CE, 2008) demonstrated that SVR after treatment with interferon, either standard or pegylated, with or without Ribavirin, is only 41%. Higher response rates were observed with higher dosing in patients without cirrhosis, with low viral loads, elevated transaminases, and genotype 1 infection [26]. In 2009, the same author provided additional data, noting that SVR is higher with an extended treatment duration among female patients and in those demonstrating an early response to treatment [27]. Controversially, a meta-analysis of over 650 HD patients treated for HCV with either standard or pegylated interferon (Alavian SM and Tabatabaei SV, 2010) indicate that the only characteristic correlating with SVR in these patients is an age under 40 at the time of antiviral treatment [28]. Another meta-analysis (Fabrizi F. 2014) highlights the dropout rate of 0.18% in HCV patients undergoing HD treated with interferon and Ribavirin, anemia and infections being the main reasons [29]. Subsequently, the same author reveals that HCV seropositivity in HD patients is an independent risk factor for overall mortality, particularly from hepatic and cardiovascular causes [24]. As a conclusion, another meta-analysis (2020), not limited to HD patients, proves the impact of HCV on the onset and progression of CKD, as well as the beneficial effect of HCV treatment in this context [43].

(b) DAA treatment meta-analysis. Our research identified several other meta-analyses that address the outcomes of sofosbuvir-based therapeutic regimens. A meta-analysis including over 700 patients with stage 4–5 CKD, more than 50% in HD (Li M, 2019), treated with sofosbuvir-based regimens demonstrated the efficacy of standard and reduced doses in these patients [35]. Shehadeh F (2020), through the results of his meta-analysis, supports the safety of sofosbuvir administration in HD patients, also mentioning the dosages used in the included studies and the incidence of adverse effects: fatigue (16%), anemia (15%), and gastrointestinal intolerance (14%) [44]. Fabrizi F. (2021) examines the impact of DAA treatment on the progression of HCV-positive patients with stage 4 and 5 CKD. He demonstrates that a 12-week sofosbuvir-based regimen, a drug primarily eliminated by the kidneys, is both safe and effective for these patients, provided that renal function is carefully monitored throughout the treatment period [45]. Prabhu RA (2023) conducted an update of 13 studies focusing on HCV treatment exclusively in HD patients, concluding that pegylated interferon is more effective than standard IFN in these patients, though the response is not well supported. Direct-acting antivirals have now replaced the use of interferons for treatment. There is insufficient high-quality data to support the efficacy of Grazoprevir, Elbasvir, and Telaprevir in this patient population [45].

**B. STUDIES REGARDING THE PROGRESSION OF HCV INFECTION IN HD**

In addition to these meta-analyses, the PubMed search yielded several other studies concerning the progression of HCV infection in HD (Table 11).

Studies around the 2000s present sometimes contradictory data. A prospective study (1996) demonstrated that over 80% of HCV-seropositive patients in HD remained carriers, and the liver biopsy proves changes only in viremic patients [41]. Conversely, another study (Okuda K, 2004) found that chronic hepatitis C in HD patients exhibits mild disease activity, and that HCV is frequently cleared in asymptomatic dialysis patients over a prolonged period [42].

The negative impact of HCV seropositivity in HD patients has been shown in several studies. Zaki MSE (2017) found that HCV has a significant impact on the development of cardiovascular diseases and on structural heart changes [56]. A meta-analysis (Fabrizi F, 2004) along with other studies (Baid-Agrawal S, (2008), Kwon E (2015), Goodkin DA (2013, 2019)) showed that HCV-positive patients on dialysis have an increased risk of general mortality, of mortality caused by hepatocellular carcinoma and liver cirrhosis, of hospitalization, anemic complications, and lower quality of life scores [47,48,52,54,55]. As a consequence of the negative impact of HCV, Sawinski D (2019) noted that HCV-seropositive patients experience reduced access to the kidney transplantation waitlist, despite gaining a substantial survival benefit from transplantation [53]. Another meta-analysis (Greeviroj P, 2022) concluded that the pooled all-cause mortality, infection-related mortality, and malignancy-related mortality are higher in HCV-positive patients than in HCV-negative patients undergoing hemodialysis. However, it was not found to be a significant difference in HCV-associated all-cause hospitalization among hemodialysis patients or in unadjusted HCV-associated cardiovascular mortality [3].

**C. STUDIES REGARDING DAA TREATMENT IN HD PATIENTS**

We also found numerous studies addressing DAA treatment in HCV(+) patients undergoing HD therapy (Table 12).

The first studies on the efficacy of DAAs in the HD population were published in 2015 and included drugs primarily eliminated by the liver. Roth D (2015), Choi DT (2019), and Liu CH demonstrated the efficacy of protocols based on the use of EBR/GZR for treating HCV genotype 1 infection in HD patients [59,71,72]. Lee BS highlighted the efficacy and safety of Daclatasvir and Asunaprevir in patients with HCV genotype 1b infection on hemodialysis [61]. The PrO ± D combination has proven effective and safe in several clinical studies targeting genotype 1 (Sato K, Sperl J, Yaraş S) [62,64,68].

**Figure 4:**
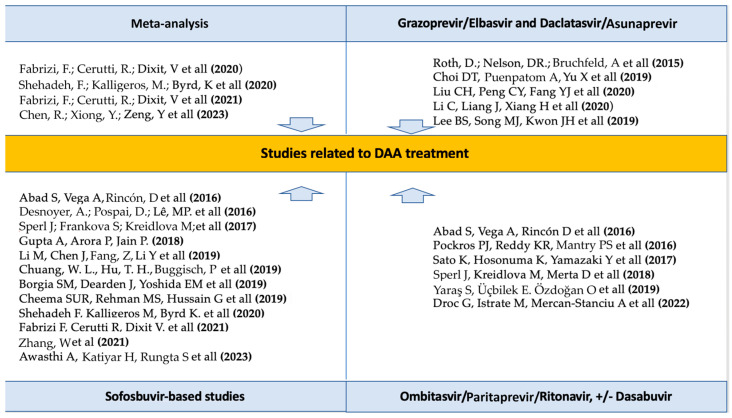
The types of studies related to DAA treatment [35,39,40,41,42,44,59,60,61,62,63,64,65,66,67,68,70,71,72,73,74,77,78].

Following the introduction of pangenotypic sofosbuvir-based regimens, the interest in administering these treatments to HD patients increased, despite initial concerns about their renal elimination. Numerous studies have demonstrated the efficacy and safety of sofosbuvir-based regimens in this patient category. (Figure 4). Over time, the administration of sofosbuvir in combination with ledipasvir or velpatasvir has been evaluated in patients with CKD, showing good efficacy and safety during the assessment period [66,69,78].

All these studies demonstrate the efficacy and safety of administering this therapy, though they do not extend the observational spectrum beyond the treatment period [60,61,73,74,75,76,77]. Furthermore, although sofosbuvir has been contraindicated for use in patients on HD due to renal elimination and potential toxicity, many studies support its utility in these patients (Figure 4).

## 4. Discussion

The prevalence of HCV in the HD population is significant, and published meta-analyses focus on the following: an assessment of the tolerability and efficacy of the antiviral treatment, either with interferon [26,27,28,29] or DAAs [39,40,41,42,43,44], focused on the treatment period; the progression of HCV(+) infection in HD patients, especially before the introduction of DAA therapy [3,45,46,47,48,49,50,51,52,53,54,55,56,57,58]; and the tolerability and efficacy of DAA therapy in HD patients. Still, there is limited reference to the long-term impact of DAA antivirals in HCV(+) HD patients, beyond the treatment period [39,44,60,61,62,63,64,65,66,67,68,69,70,71,72,73,74,75,76,77,78,79].

HCV infection is a substantial public health concern for hemodialysis patients and contributes to liver dysfunction, in addition to other pathological conditions commonly linked with chronic kidney disease (CKD) [80]. In contrast to other viral diseases that have a low incidence yet significant short-term effects on this population, HCV is characterized by a substantial prevalence and a well-documented influence on long-term survival [81]. The development of DAA therapies decreased the prevalence data over time [82,83]. HCV infection is endemic in our country and in our region; the prevalence of HCV seropositivity in hemodialysis patients found in our study, throughout the monitoring period, is 2.56%. This result can be attributed to the restricted patient cohort in the study, their advanced age resulting in elevated mortality rates, and the possible underdiagnosis of HCV in the early 2000s when screening was only aimed at individuals exhibiting suggestive clinical and biological alterations. The reuse of dialyzers was implemented for a restricted duration until the 2000s, with each patient possessing a designated dialysis set marked for exclusive personal usage. The annual incidence of HCV in our hemodialysis centers during the past two years was 1.8%. No new HCV seropositivity was recorded after the onset of hemodialysis. It has been shown that the primary risk factors for HCV infection in hemodialysis patients include a younger age (under 50 years at the initiation of hemodialysis), prolonged duration of hemodialysis, transfusion needs, and the reuse of dialyzers. In our analysis, the mean age of HCV seropositive patients was 66,972 years (median age 70 years), similar to the control group (64,784 years, *p* = 0.403), with a prevalence of males in both patient cohorts (59.2% vs. 54.9%). Despite HCV-positive patients being older than the control group, no clinical or biological results were documented in comparison to those of HCV-negative patients. The duration of hemodialysis was comparable between the two groups, with an identical median of 7 years (8.85 years for HCV-positive patients and 8.1 years for HCV-negative patients). No significant differences were seen between HCV-positive and -negative individuals for the likelihood of a prior kidney transplant (*p* = 0.22). When exclusively comparing HCV-treated patients to HCV-negative individuals, the proportion reached statistical significance (17.39% vs. 4.5%, *p* = 0.02). This is due to the explicit desire from transplant hospitals to achieve viral clearance before the procedure.

AVF is the main vascular access for dialysis in both patient groups, with no statistically significant differences noted (68.5% vs. 75.6%, *p* = 0.430). The extracorporeal manipulation of blood enhances the risk of HCV transmission due to increased exposure to the environment, particularly when disinfection procedures are inadequate. Nowadays, the necessity for pre-dialysis transfusions has significantly diminished due to the use of erythropoiesis-stimulating drugs (ESAs), rendering nosocomial transmission the primary method of HCV transmission in HD [84]. HCV can survive for up to 16 h in external environments, including on dialysis machines and medical equipment. Without adequate disinfection, both CVCs and AVF may favor the entrance of HCV into the bloodstream [85]. Chronic skin lesions of the lower limbs may also serve as entry points of HCV [86]. On the other side, HCV infection increases the incidence of bacteremia in patients dialyzed on long-term catheters, diminishing long-term survival [87].

In hemodialysis patients, the consequences of HCV infection are not well understood, and its progression is notably challenging to ascertain. The clinical manifestation of the disease is nonspecific and ambiguous. The diagnosis of HCV infection may be inadequate due to the failure to identify anti-HCV antibodies using low-specificity tests in asymptomatic individuals. The mortality rate due to non-hepatic causes in these patients is several times higher than in non-uremic patients, making it challenging to assess the long-term consequences of this infection.

The presence of HCV infection continuously stimulate the immune system, which in turn might lead to a variety of immune-mediated pathologies and the disruption of various metabolic processes [88,89]. However, in patients undergoing HD treatments, this situation is counterbalanced by a blunted immune response. So, these patients exhibit clinically silent forms of hepatitis, with a slow progression to cirrhosis or hepatocellular carcinoma, lower levels of transaminases, and subtle changes in liver biopsy results [47]. This explains why, in our patients, at a similar efficacy of HD reflected by eKT/V, we did not identify statistically significant differences in biometric data (both BMI and BSA) or laboratory analyses when compared to the HCV-negative population [90,91,92,93,94].

Among patients with a similar duration of HD, the levels of serum hemoglobin, inflammatory syndrome quantified by CRP, liver enzymes, and parameters of bone mineral balance did not differ significantly, regardless of the presence or absence of HCV. Unfortunately, the data regarding liver fibrosis were missing in our patients. The gold standard to stage hepatic fibrosis is percutaneous liver biopsy. However, it is an invasive procedure with poor patient acceptance. Currently, there are biochemical, serological, and radiological noninvasive indices to predict the stage of hepatic fibrosis. Transient Elastography (TE, FibroScan), directly measuring liver stiffness by sonography-based techniques, can diagnose the severity of hepatic fibrosis, avoiding a liver biopsy in up to 90% of hemodialysis patients with HCV infection [95]. If TE is unavailable, the AST-to-Platelet Ratio Index (APRI), King’s Score, and Fibrosis Index Based on Four Parameters (FIB-4) might be used, keeping in mind that the cut-off values to stage hepatic fibrosis are lower in hemodialysis patients than in nonuremic patients. Because most hemodialysis patients present with systemic inflammation/fibrosis from nonhepatic origins, serological indices (FibroTest, hyaluronic acid, and Tyrosine–Lysine–Leucine 40 Kilodalton YKL-40) are not recommended in clinical practice to predict the stage of hepatic fibrosis in HD patients with HCV infections. Moreover, the noninvasive procedures are not able to monitor the fibrosis evolution in HD patients [96].

In our HD patients, we did not identify statistically significant differences regarding associated pathologies. HCV represents a risk factor for the development of hypertension, diabetes mellitus, coronary artery disease requiring interventional cardiology, and cerebrovascular accidents [24,54,97,98]. In our study, cardiovascular disease had a similar prevalence, regardless of the presence of HCV, but among patients receiving anti-HCV treatment (DAA), the percentage of hypertension was lower compared to HCV-negative patients (*p* = 0.0502).

Malignancy was the only pathology that showed a statistically significant higher incidence in patients tested positive for HCV. The role of viruses in the development of cancer is now widely acknowledged. HCV is recognized as an oncogenic virus linked to human cancers, in conjunction with human papillomavirus (HPV), Epstein–Barr virus (EBV), human herpes virus 8 (HHV8), Merkel cell polyomavirus (MCPyV), human T-lymphotropic virus (HTLV-1), and hepatitis B virus (HBV) [99]. HCV may promote the occurrence of hepatocellular carcinoma (HCC) through several mechanisms: (1) it creates a persistent proliferative and anti-apoptotic signaling milieu, (2) it alters signaling pathways associated with differentiation, adhesion, and angiogenesis, and (3) HCV influences signaling pathways pertinent to the inflammatory response [94]. Hepatocellular carcinoma is a significant concern worldwide, as it holds the second position in cancer-related deaths, and the available treatment modalities are quite limited [100]. Within the examined cohort, hepatocellular carcinoma was noticed in only one patient with a long history of both dialysis and HCV infection, occurring roughly two years post-treatment with DAAs. Moreover, especially in patients with severe hepatic impairment, the risk of developing HCC after treatment with DAAs remains elevated. The spectrum of malignancies in our HCV-positive patients was rather broad, including basal cell carcinoma, sarcoma, pancreatic neoplasm, and pulmonary neoplasm, all manifesting clinically after DAA treatment. However, it is hazardous to establish a correlation between the occurrence of these cancers and the presence of HCV infection or DAA treatment, considering the small population sample. When comparing HCV(+) patients treated with DAAs to untreated HCV(+) patients, the incidence of malignancies was found to be similar. Factors such as age, comorbidities, duration of HCV infection, and treatment history are potential confounders that can all influence cancer risk and mortality outcomes. Furthermore, the prevalence of cancers in patients with CKD in our country is high [101]. Nevertheless, the high statistical significance of the occurrence of this pathology in the context of HCV infection among the HD population, with clinical onset following sustained viral clearance achieved through DAA therapy, raises questions and suspicions that require clarification over time.

After DAAs became available for the treatment of HD patients, there was considerable enthusiasm for their use among all HCV-viremic patients. As treatment guidelines for HD patients focused solely on the type of therapy for different viral genotypes and its duration, the selection of patients for treatment was random, dictated by clinical status and the explicit wishes of the patients. Given the proven increase in transplantation rates following HCV eradication and the favorable long-term outcomes post-transplantation, a strong indication for treatment should be directed towards viremic patients on waiting lists for renal transplantation [57]. Numerous studies have revealed that DAAs have a significant impact on liver stiffness kinetics, particularly within the first year post-treatment, followed by stabilization [102,103]. However, the long-term risk of cirrhosis decompensation remains elevated following DAA treatment, but data in this regard are limited in HD patients [104].

Patients with HCV infection have a distinct psychological profile. All tests applied to assess depression, life satisfaction, and well-being show unfavorable results for HD HCV-seropositive patients. This situation is attributed not only to their poor biological condition, which is similar in both groups, but also to the presence of HCV in the central nervous system (CNS), leading to morpho-functional changes [105]. The entry of HCV into the CNS occurs through peripheral blood mononuclear cells, which act as a Trojan horse [106,107]. Several pathogenetic mechanisms underlying neurological symptoms have been hypothesized, including neuro-invasion, immune-mediated damage, neurotransmitter alterations, and cryoglobulinemia. Alterations of the CNS may include cerebral vasculopathy, acute or subacute encephalopathy, and inflammatory disorders [108,109]. A significant number of patients infected with HCV exhibit neuropsychiatric symptoms such as “brain fog,” fatigue, and a diminished quality of life, independent of the severity of their liver disease. The quality of life for these patients is adversely affected by anxiety and depression [105,110].

This study has several limitations: it is a retrospective, observational study conducted on a relatively small number of patients; it is aimed at Romanian residents, so it is not clear whether this nomogram model is applicable to other races; data regarding viral load were available only for patients who underwent antiviral treatment; liver biopsy was not performed in any case, and neither was TE; the presence of HCV was assessed through immuno-enzymatic methods, which may have contributed to underdiagnosis; and data collection was performed at the same time for living patients and at their last presentation in the center for deceased patients, which could lead to potential discrepancies in laboratory values. Despite these limitations, the study raises an alarm and invites the conduct of large-scale, multinational clinical studies to illustrate the evolution of HCV-seropositive patients undergoing hemodialysis in the DAA era and to specify firm treatment indications for this population.

## 5. Conclusions

The natural history of HCV infection in HD patients remains insufficiently elucidated. Given the silent clinical picture, reduced biological resonance, and decreased ability to evaluate liver fibrosis in a noninvasive way, the pathogenesis of this condition is still not fully understood. With the introduction of DAA treatment, particularly pangenotypic coverage, sustained viral clearance has been achieved in HD patients. However, the long-term evolution post-DAA treatment has yet to be thoroughly evaluated. Malignancy represents identifiable complications and serves as a reliable endpoint in the progression of HCV-infected hemodialysis patients. Further studies are needed to investigate the impact of HCV infection and DAA treatment on oncogenesis.

## Figures and Tables

**Table 1:** General characteristics of HCV(+) and HCV(−) population.

General Characteristics	HCV(+) (*n* = 54)	HCV(−) (*n* = 111)	*p*-Value
Age (M ± SD)	66.972 ± 11.569	64.784 ± 13.694	*p* = 0.403 ^†^ *p* = 0.402 ^¥^
Gender (nr/%) Male	32 (59.2)	60(54.05)	*p* = 0.6008 ^‡^
Time on HD (years) (M/Me)	8.852 ± 7.718 7	8.099 ± 5.346 7	*p* = 0.628 ^†^ *p* = 0.627 ^¥^
Previous transplantation%	5 9.25%	4 3.6%	*p* = 0.2297 ^‡^
Type of vascular access(nr/%) CVC AVF	17 (31.48) 37 (68.5)	27(24.3) 84 (75.6)	*p* = 0.4307 ^‡^
kT/V	1.564 ± 0.337 1.59	1.550 ± 0.318 1.61	*p* = 0.314 ^†^ *p* = 0.313 ^¥^
Dry weight (kg)M ± SD Me	69.47 ± 23.79 67.75	71.55 ± 17.27 72.5	*p* = 0.2708 ^†^
BMI (kg/m^2^)	25.070 ± 6.905	25,419 ± 5.579	*p* = 0.431 ^†^
BSA M ± SD Me	1.81 ± 0.32 1.78	1.82 ± 0.24 1.84	*p* = 0.3836 ^†^

AVF = arteriovenous fistula; BMI = body index mass; BSA = body surface mass; CVC = central venous catheter; M = mean; Me = median; SD = standard deviation; ^†^ Mann–Whitney test; ^‡^ = Fisher test; ^¥^ = Kruskal–Wallis.

**Table 2:** Laboratory findings regarding HCV seropositivity.

Laboratory Parameters (M ± SD/Me)	HCV(+)	HCV(−)	Statistical Significance Mann–Whitney Test ^†^
Hemoglobin (g/dL)	10.572 ± 1.640 10.85	10.662 ± 1.455 10.8	*p* = 0.823 ^†^ *p* = 0.821 ^¥^
CRP (mg/L)	28.979 ± 46.958 15	30.877 ± 41.316 13.3	*p* = 0.784 ^†^ *p* = 0.782 ^¥^
Thrombocytes ×10^3^/mm^3^	204 ± 82.65 196	217.14 ± 84.6 212	*p* = 0.191 ^†^
ALT (IU/L)	25.574 ± 25.844	22.586 ± 17.597	*p* = 0.530 ^†^ *p* = 0.529 ^¥^
Alkaline phosphatase (IU/L) M/Me	106.22 96	120.83 91	*p* = 0.235 ^†^
Serum albumin (g/dL)	3.676 ± 0.508 3.7	3.804 ± 0.366 3.9	*p* = 0.154 ^†^ *p* = 0.153 ^¥^
iPTH (pg/mL)	413.096 ± 464.566 204	397.225 ± 448.846 275	*p* = 0.801 ^†^ *p* = 0.800 ^¥^
Phosphate (mg/dL)	4.998 ± 1.619 4.7	4.946 ± 1.809 4.8	*p* = 0.876 ^†^ *p* = 0.874 ^¥^
Total calcium (mg/dL)	8.913 ± 0.982 9	8.865 ± 0.0825 8.9	*p* = 0.634 ^†^ *p* = 0.633 ^¥^
Total cholesterol (mg/dL)	159.167 ± 50.103 141	163.946 ± 46.162 148.5	*p* = 0.616 ^†^ *p* = 0.615 ^¥^
Serum bicarbonate (mEq/L)	24.480 ± 2.926 25	24.273 ± 4.074 24.5	*p* = 0.508 ^†^ *p* = 0.507 ^¥^
Creatinine (mg/dL)	7.277 ± 2.425 7.2	8.18 ± 5.88 7.2	*p* = 0.163 ^†^

^¥^ = Kruskal–Wallis, Mann–Whitney Test ^†^.

**Table 3:** Prevalence of major comorbidities in the studied HD population.

Comorbidities	HCV(+) (*n* = 54)	HCV(−) (*n* = 111)	OR	*p*-Value ^‡^
Arterial hypertension	45 (83.3%)	104 (93.6%)	0.34	*p* = 0.067
Diabetes mellitus	11(20.3%)	26(23.4%)	0.84	*p* = 0.659
Severe coronary disease (DES)	19 (35.1%)	40 (36.03%)	0.96	*p* = 0.914
Stroke	9 (16.6%)	12(10.8%)	1.65	*p* = 0.289
Neoplasia	11(20.3%)	9(8.1%)	2.96	*p* = 0.023
Causes of death neoplasia cardiac reasons stroke others	N (29) (53.7%) 4 (13.8%) 20 (68.9%) 1 (3.4%) 4 (13.8%)	N (52) (47.74%) 2 (3.77%) 36 (67.92%) 2 (3.77%) 13 (24.52%)	4.36 1.23 1.03 0.6	*p* = 0.091 *p* = 0.550
Age at the time of death M ± SD/Me	67.35 ± 13.58 70	70.76 ± 10.05 70.5		*p* = 0.079

^‡^ = Fisher test.

**Table 4:** Demographics and laboratory findings after DAA treatment.

	HCV(+) (DAA tr) (*n* = 23)	HCV(−) (*n* = 111)	Statistical Significance († Mann–Whitney Test)
Age (years)	63.174 ± 11.203	64.784 ± 13.694	*p* = 0.480
Gender (m)	14	60	*p* = 0.536 * p = 0.768 ^‡^
Time in HD	10.391 ± 7.703	8.099 ± 5.346	*p* = 0.361 ^†^
BMI (kg/m^2^)	26.870 ± 7.272	23.468 ± 6.434	*p* = 0.063 ^†^
Laboratory parameters (M ± SD)/Me	HCV+ (DAA tr)	HCV(−)	
Hemoglobin	10.674 ± 1.667 10.7	10.51 ± 1.675 10.8	*p* = 0.4329 ^†^
kT/V	1.596 ± 0.378 1.58	1.550 ± 0.318 1.61	*p* = 0.893 ^†^
CRP (mg/L)	33.17 ± 66.07 12.8	30.09 ± 42.05 13.3	*p* = 0.4111 ^†^
Thrombocytes ×10^3^/mm^3^	212.32 ± 100.79 211	217.14 ± 84.6 212	*p* = 0.4129 ^†^
ALT(IU/L)	22.261 ± 19.163 18	29.107 ± 31.280 17	*p* = 0.105 ^†^
AST(IU/L)	18.65 ± 10.9 19	21.78 ± 17.43 16	*p* = 0.2053 ^†^
Alkaline phosphatase (IU/L)	92.41 ± 53.42 89	120.83 ± 121.62 91	*p* = 0.1742 ^†^
Serum albumin (g/dL)	3.649 ± 0.599 3.6	3.691 ± 0.456 3.9	*p* = 0.947 ^†^
iPTH (pg/mL)	281.052 ± 269.422 187	431.143 ± 413.597 275	*p* = 0.264 ^†^
Phosphate (mg/dL)	4.897 ± 1.644 5.1	4.766 ± 1.269 4.8	*p* = 0.947 ^†^
Total calcium (mg/dL)	8.913 ± 1.307 8.6	8.879 ± 0.669 8.9	*p* = 0.663 ^†^
Total cholesterol (mg/dL)	170.174 ± 56.234 149	155.607 ± 42.463 148.5	*p* = 0.298 ^†^
Serum bicarbonate (mEq/L)	24.335 ± 3.489 23.9	24.911 ± 2.318 24.5	*p* = 0.555 ^†^
Creatinine (mg/dL)	7.94 ± 2.72 7.76	8.18 ± 5.88 7.2	*p* = 0.4494 ^†^

* = Pearson chi2; ^‡^ = Fisher, ^†^ Mann–Whitney Test.

**Table 5:** Comorbidities after DAA treatment.

Comorbidities	HCV(DAA-Tr) (*n* = 23)	HCV (−) (*n* = 111)	*p*-Value x^2^ = Chi Test (^‡^ = Fisher) (^¥^ = Kruskal–Wallis)
Previous Tx	4 (17.39)	5 (4.5%)	*p* = 0.0246 **^‡^**
Arterial hypertension	18 (78.2%)	104(93.6%)	*p* = 0.0502 ^¥^
Diabetes mellitus	5(21.73)	26(23.4)	*p* = 0.5276 x^2^
Severe coronary disease (DES)	6 (26)	40 (36.03)	*p* = 0.3603 x^2^
Stroke	3(13.04)	12(10.8)	*p* = 0.7572 x^2^
Neoplastic diseases	7 (30.43)	9 (8.1)	*p* = 0.0079 x^2^
Main causes of death neoplasia cardiac reasons	*n* = 8 4 (50%) 3 (37.5%)	*n* = 52 2 (3.84) 39 (75%)	*p* = 0.0010 ^‡^ *p* = 0.0240 ^¥^
Age at the time of death (M ± SD/Me)	57.63 ± 15.18 58.5	70.76 ± 10.05 70.5	*p* = 0.0009 x^2^

**Table 6:** Demographics, laboratory, and comorbidities after DAA treatment compared to HCV(+)nTr.

Comorbidities	HCV(DAA-Tr) (*n* = 23)	HCV(+)nTr (*n* = 28)	*p*-Value
Age (years)	63.174 ± 11.203	69.893 ± 11.259	*p* = 0.041 ^¥^
Gender (male)	14	14	*p* = 0.57 ^‡^
Previous Tx	4 (17.39)	5 (4.5%)	*p* = 0.0246 ^‡^
ALAT (IU/L)	29.107 ± 31.280	22.261 ± 19.163	*p* = 0.027 ^¥^
Cholesterol (mg/dL)	155.607 42.463	170.174 56.234	*p* = 0.040 ^¥^
Arterial hypertension	19 (82.6%)	27 (96.42%)	*p* = 0.099 * p = 0.120 ^‡^
Diabetes mellitus	5 (21.73)	6 (21.42%)	*p* = 0.967 * p = 0.62 ^‡^
Severe coronary disease (DES)	8 (34.78%)	11 (39.28%)	*p* = 0.741 * p = 0.485 ^‡^
Neoplastic diseases	7 (30.43)	4 (14.28%)	*p* = 0.190 * p = 0.146 ^‡^
Age at the time of death (M ± SD/Me)	57.63 ± 15.18 58.5	69.893 ± 11.259	*p* = 0.041 ^¥^

* x^2^ Chi test; ^‡^ = Fisher test; ^¥^ = Kruskal–Wallis test.

**Table 7:** General characteristics of studied population; laboratory findings.

Parameter M ± SD	All Patients (165)	HCV(+) nTr (28)	HCV(+) DAA (23)	HCV(+) Ifn Tr (3)	HCV(−) (111)	*p*-Value (Kruskal–Wallis)
Age (years)	65.500 ± 13.042	69.893 ± 11.259	63.174 ± 11.203	68.833 ± 12.82	64.784 ± 13.694	*p* = 0.187
Gender (M) n/%	92 55.75%	14 9.09%	14 8.48%	3 1.81%	60 36.36%	*p* = 0.249 * p = 0.277 ^‡^
Previous Tx n/%	9 5.45%	1 0.6%	4 0.24%	0	4 0.24%	*p* = 0.66 *
Vascular access (AVF n/%)	121 73.33%	19 11.51%	17 10.3%	1 0.6%	84 50.9%	*p* = 0.679 ^‡^
Time in HD M ± SD	8.345 ± 6.208	7.964 ± 7.965	10.391 ± 7.703	5.333 ± 3.512	8.099 ± 5.346	*p* = 0.323 ^¥^
BMI (kg/m^2^) M ± SD		23.468 ± 6.434	26.870 ± 7.272	26.233 ± 6.917	25.419 ± 5.579	*p* = 0.86^¥^
KT/V	1.56 ± 0.35	1.550 ± 0.318	1.596 ± 0.378	1.453 ± 0.206	1.61 ± 0.36	*p* = 0.671 ^¥^
Hb (g/dl)	10.571 ± 1.667	10.510 ± 1.675	10.674 ± 1.667	10.367 ± 1.604	10.662 ± 1.455	*p* = 0.972 ^¥^
ALT (IU/l)	23.564 ± 20.628	29.107 ± 31.280	22.261 ± 19.163	18.000 ± 8.000	22.586 ± 17.597	*p* = 0.142 ^¥^
Albumin (g/dl)	3.762 ± 0.421	3.691 ± 0.456	3.649 ± 0.599	3.730 ± 0.279	3.804 ± 0.367	*p* = 0.557 ^¥^
T chol mg/dL	162.382 ± 47.388	155.607 ± 42.463	170.174 ± 56.34	108.000 ± 43.578	163.946 ± 46.162	*p* = 0.193 ^¥^
iPTH (pg/mL)	402.419 452.693	431.143 ± 413.597	281.052 ± 269.422	1257.000 ± 1156.780	397.225 ± 448.846	*p* = 0.205 ^¥^
			281.052 ± 269.422	1257.000 ± 1156.780		*p* = 0.046 ^¥^
Ca (mg/dL)	8.881 ± 0.877	8.879 ± 0.669	8.913 ± 1.307	9.233 ± 0.751	8.865 ± 0.825	*p* = 0.816 ^¥^
P (mg/dL)	4.963 ± 1.744	4.766 ± 1.269	4.897 ± 1.644	7.927 ± 2.064	4.946 ± 1.809	*p* = 0.132 ^¥^
		4.766 ± 1.269		7.927 ± 2.064		*p* = 0.022 ^¥^
			4.897 ± 1.644	7.927 ± 2.064		*p* = 0.021 ^¥^
Bicarbonate	24.341 ± 3.730	24.911 ± 2.318	24.335 ± 3.489	21.567 ± 2.205	24.273 ± 4.074	*p* = 0.31 ^¥^
CRP (mg/dL)	30.256 ± 43.108	28.825 ± 32.281	29.090 ± 62.335	29.567 ± 40.281	30.877 ± 41.316	*p* = 0.846 ^¥^

* x^2^ Chi test; ^‡^ = Fisher test; ^¥^ = Kruskal–Wallis test.

**Table 8:** Prevalence of major comorbidities in the studied HD population related to HCV status.

Parameter		All Patients (*n* = 165)	HCV(+) DAA (*n* = 26)	HCV(+) Ifn (*n* = 3)	HCV(+) nTr (*n* = 28)	HCV(−) (*n* = 111)	*p*-Value
Diabetes mellitus n/%		38 (23.03%)	5 (19.23)	1 (33.33%)	6 (21.42%)	26 (23.42%)	*p* = 0.982 ^‡^
Arterial hypertension n/%		152 (92.12%)	19 (73.03)	3 100%	27 (96.42%)	103 (92.79%)	*p* = 0.416 ^‡^
Cardivascular diseases (DES)n/%		62 (37.57%)	8 (34.78%)	2 (66.66%)	11 (39.28%)	41 (36.93%)	*p* = 0.807 ^‡^
Neoplastic diseases n/%		20 (12.12%)	7 (26.92%)	0	4 (14,28%)	9 (8.1%)	*p* = 0.024 * p = 0.028 ^‡^
Alive status (deaths) n/%		81 (49.09%)	10 (38.46%)	0	19 (67.85%)	52 (46.84%)	
Causes of death (*n* = 81)	Cardiovascular	62 (76.54%)	5 (50%)	-	17 (89.47%)	40 (76.92%)	*p* = 0.22 ^¥^
	infections	8 (9.87%)	-	-	1 (5.26%)	7 (13.46%)	*p* = 0.74 ^¥^
	neoplasis	6 (7.4%)	4 (40%)	-	-	2 (3.84%)	*p* = 0.17 ^¥^
	miscellaneous	5 (6.17%)	1 (10%)	-	1 (0%)	3 (5.76)	-

* = x^2^ Chi test; ^‡^ = Fisher; ^¥^ = Kruskal–Wallis.

**Table 9:** Tests applied to evaluate psychological conditions associated with HCV infection.

Survey	Patients Groups	Subjects nr	Mean Ranks	*p*-Value
BDI	HCV(+)	46	34	*p* < 0.00001
	HCV(−)	78	19.76	
PWS	HCV(+)	46	53.52	*p* < 0.00001
	HCV(−)	78	59.76	
TSWLS	HCV(+)	46	53.2	*p* < 0.00001
	HCV(−)	78	69.69	
SF-36	HCV(+)	46	50.67	*p* < 0.00001
	HCV(−)	78	61.32	

**Table 10:** The main meta-analyses regarding anti-HCV treatment.

	Meta-Analysis Regarding HCV Treatment in HD	Type of Treatment
1	Gordon CE, Uhlig K, Lau J et al. (2008) [26]	Interferon treatment
2	Gordon CE, Uhlig K, Lau J et al. (2009) [27]	
3	Alavian SM, Tabatabaei SV (2010) [28]	
4	Fabrizi F, Dixit V, Messa P et al. (2014) [29]	
1	Fabrizi F, Lampertico P, Messa P. (2018) [41]	DAA treatment
2	Li M, Chen J, Fang Z, Li Y et al. (2019) [42]	
3	Fabrizi F, Dixit V, Messa P (2019) [24]	
4	Fabrizi F, Cerutti R, Dixit V et al. (2020) [43]	
5	Shehadeh F, Kalligeros M, Byrd K et al. (2020) [44]	
6	Fabrizi F, Cerutti R, Dixit V et al. (2021) [45]	
7	Prabhu RA, Nair S, Pai G et al. (2023) [46]	
8	Chen R, Xiong Y, Zeng Y et al. (2023) [47]	

**Table 11:** PubMed monitoring studies.

Study	No of Patients	Duration (Years)	Study Type
Yamaji K, Hayashi J, Kawakami Y et al. (1996) [48]	115	5	Pr
Okuda K, Yokosuka O (2004) [21]	189	18	CC
Fabrizi F, Martin P, Dixit V et al. (2004) [49]	2341	n/a	MA
Baid-Agrawal S, Pascual M, Moradpour D et al. (2008) [50]	68	12 w	
Alsaran KA, Sabry AA, Alghareeb AH et al. (2009) [51]	83	1	RCC
Goodkin DA, Bieber B, Gillespie B et al. (2013) [52]	4735	5	Ob
Wang KL, Xing HQ, Zhao H et al. (2014) [53]	19	2	Pr
Kwon E, Cho JH, Jang HM et al. (2015) [54]	123	n/a	PrCH
Goodkin DA, Bieber B, Jadoul M et al. (2017) [55]	5751	20	PrCH
Zaki MSE (2017) [56]	60	1	CC
Sawinski D, Forde KA, Lo Re V 3rd et al. (2019) [57]	31,624	10	RCH
Saadi G, Kalantar-Zadeh K, Almasio P et al. (2020) [58]		n/a	C
Greeviroj P, Lertussavavivat T, Thongsricome T et al. (2022) [3]	125,972	32	MA

**Abbreviations:** Pr—prospective, CC—case–control, MA—meta-analysis, R—retrospective, Ob—observational study, CH—cohort study, n/a—not available.

**Table 12:** Articles regarding DAA treatment at HD patients.

Study	Nr of Patients/Viral Genotype	Duration	Study Type
Roth D et al. (2015) [57]	112/gen 1	12 w	P/RA
Pockros PJ et al. (2016) [58]	n/a	12 w	Rev
Abad S et al. (2016) [59]	29	12 w	R
Sato K et al. (2017) [60]	4	12 w	Pr
Sperl J et al. (2017) [61]	6/gen 3	12 w	R
Sperl J et al. (2018) [62]	19/gen 1	12 w	R
Gupta A et al. (2018) [63]	7	12–24 w	CH
Chuang, WL et al. (2019) [64]	48	12 w	Pr
Li M et al. (2019) [39]	418	12 w	MA
Lee BS et al. (2019) [65]	21	12 w	Pr
Yaraş S et al. (2019) [66]	25/gen 1	12 w	R
Borgia SM et al. (2019) [67]	59/gen 1–6	12 w	Pr
Cheema SUR et al. (2019) [68]	36/gen 1–3	12 w	Pr
Choi DT et al. (2019) [69]	563	12 w	R/CH
Liu CH et al. (2020) [70]	40	12 w	Pr
Li C et al. (2020) [71]	25	12–24 w	R
Zhang W et al. (2021) [72]	10	12 w	R
Liu CH (2022) [73]	-	-	Rev
Ryu JE et al. (2022). [74]	22	12 w	R
Droc G et al. (2022) [75]	108	12 w	Pr
Awasthi A S et al. (2023) [76]	880	8 w	RA
Chen R et al. (2023) [44]	2377	12 w	MA
Naguib H et al. (2024) [77]	60	12 w	CS

RA—randomized study, CS—cross-sectional.

## Data Availability

Data are contained within the article.
